# Quantitative 3D Reconstruction from Scanning Electron Microscope Images Based on Affine Camera Models

**DOI:** 10.3390/s20123598

**Published:** 2020-06-26

**Authors:** Stefan Töberg, Eduard Reithmeier

**Affiliations:** Institute of Measurement and Automatic Control, Faculty of Mechanical Engineering, Leibniz University Hannover, Nienburger Str. 17, 30167 Hannover, Germany; sekretariat@imr.uni-hannover.de

**Keywords:** scanning electron microscope, 3D reconstruction, affine camera, epipolar geometry, self calibration, rectification, triangulation, dense point cloud, registration

## Abstract

Scanning electron microscopes (SEMs) are versatile imaging devices for the micro- and nanoscale that find application in various disciplines such as the characterization of biological, mineral or mechanical specimen. Even though the specimen’s two-dimensional (2D) properties are provided by the acquired images, detailed morphological characterizations require knowledge about the three-dimensional (3D) surface structure. To overcome this limitation, a reconstruction routine is presented that allows the quantitative depth reconstruction from SEM image sequences. Based on the SEM’s imaging properties that can be well described by an affine camera, the proposed algorithms rely on the use of affine epipolar geometry, self-calibration via factorization and triangulation from dense correspondences. To yield the highest robustness and accuracy, different sub-models of the affine camera are applied to the SEM images and the obtained results are directly compared to confocal laser scanning microscope (CLSM) measurements to identify the ideal parametrization and underlying algorithms. To solve the rectification problem for stereo-pair images of an affine camera so that dense matching algorithms can be applied, existing approaches are adapted and extended to further enhance the yielded results. The evaluations of this study allow to specify the applicability of the affine camera models to SEM images and what accuracies can be expected for reconstruction routines based on self-calibration and dense matching algorithms.

## 1. Introduction

The scanning electron microscope (SEM) is a common tool that is used for analysation purposes in the fields of biomedical, mechanical and material sciences [1,2,3]. To generate images, an electron beam scans the surface of a specimen in a raster scan pattern. On the defined pattern locations that directly correspond to certain pixel positions in the image, the interaction of the high energy electrons with the material causes the specimen to emit additional electrons. To illustrate the topographical contrast of the investigated specimen, the low-energy electrons from surface-near layers are detected and further processed to greyscale values. Common detectors for this purpose are Everhart-Thornley (EVT) or newer In-Lens detectors [4]. Although the SEM image intensities illustrate the topographical contrast of the sample, they do not contain information about its depth. Even though some morphological analyses only depend on the 2D information of images, detailed characterizations include the 3D surface structure so that other measurement devices like confocal laser scanning microscopes (CLSMs) or atomic force microscopes (AFMs) have to be used instead. Therefore, different approaches were presented in the last decade to overcome this limitation and extend the SEM’s measurements to the third dimension.

Krachtovil et al. [5] acquired large sets of images by mounting specimens on nanobelts. These allow local rotations that result in views onto the specimen as if a camera rotates up to 360${}^{\circ }$ around the object which stays at the position of the rotation center. The depth reconstruction of sparse feature correspondences between neighbouring images of a sequence is performed based on batch factorization [6]. Due to the usage of sparse feature points, large amounts of images are necessary to reconstruct a meaningful representation of the imaged surface. Therefore, the necessary sample preparation and measurement process is time-consuming. An evaluation of the reconstructions, comparing them with ground truth data or other metric measurements is missing. Xie [7] analysed SEM stereo-pair images based on an eucentric tilting procedure so that corresponding points between the images undergo a nearly pure horizontal parallax during the tilting. This allows to directly compute the scene’s depth from trigonometric equations under the assumption that the projection properties of the SEM can be described by an orthographic projection model and that the tilt angle is known. Zhu et al. [8] used a calibration process to determine the intrinsic and extrinsic camera parameters for a defined stage position and fixed SEM imaging setting with a magnification in the lower range of 300. Based on this calibration and the pinhole camera model, a stereo vision approach was applied to perform strain measurements. The major drawbacks of this method are that the measurement results depend on the accuracy of the stage and that for different magnifications and imaging settings new calibrations are needed. Tafti et al. used a non-linear optimization method to determine the stage’s motion [9]. The authors refer to the use of projective geometry to determine an initial estimate. Subsequently, the structure is triangulated from sparse feature correspondences and a surface mesh is generated. Baghaie et al. [10,11] determine a dense reconstruction applying algorithms that are partly linked to the perspective and orthographic projection model. The images are rectified based on the general pinhole camera model to perform a row-wise search for dense correspondences. From the obtained horizontal parallax between the rectified images, the depth is directly computed by trigonometry according to Xie [7], relying on the orthographic projection properties. Kudryavtsev et al. [12] estimate the rotation angles without any prior knowledge of the stage’s motion using Shimshoni’s interpretation of the affine camera [13]. Shimshoni described the affine camera center lying on a plane at infinity so that the camera motion can be described by rotations which angles can be computed using affine epipolar geometry of neighbouring image pairs and spherical trigonometry. Nevertheless, this approach is not ideal since the camera positions are restricted to form a triangle. Additionally, it behaves less accurate in the presence of Gaussian noise compared to factorization based approaches that simultaneously minimize the geometric error in the image coordinate measurements over all used images. Kudryavtsev et al. [14] additionally evaluated a rectification method based on the affine epipolar geometry that works well for high magnification settings and smaller view changes. Nevertheless, not all parameters provided by the affine epipolar geometry of a stereo-pair are taken into account so that the approach can still be extended to improve its robustness and accuracy.

Although a lot of different approaches have been presented [5,7,10,11,14] that are capable of visualizing the 3D structure from SEM images, comparisons to other 3D measurements methods and accuracy estimations are missing. In addition, there are partly contrary studies to what extent the imaging properties of the SEM can be modelled properly by an affine camera with parallel projection rays. Cui et al. compared the affine and projective camera models based on a 2D calibration pattern for different magnification settings [15]. They examined magnifications between 300 and 10,000, concluding that the affine projection model has a comparable performance to the perspective one in terms of the obtained reprojection errors for detected ground points. Ritter et al. [16] used a 3D calibration pattern and the affine camera model to successfully calibrate the positions of a stage. Both studies state that the consideration of image distortions does not alter the calibration results significantly. Although some approaches made use of applying the perspective projection model to SEM images successfully [8,10,15], overparameterized models tend to be susceptible to noise and corrupted data. When the perspective effects diminish, the simplified affine camera models are not only a good approximation of the perspective counterpart, their application is even more suitable and results in higher robustness and accuracy [17]. Taking the ambiguities that are caused by the parallel projection rays into account, the computation of ill-conditioned parameters is avoided making the applied algorithms more stable.

Based on these outlined considerations, this study aims to give a detailed overview of the possibilities granted by the application of the affine camera model to SEM images. To yield the highest robustness and reconstruction accuracy, different sub-models of the affine camera are applied to the SEM images in the context of self-calibration to identify the model’s ideal parametrization and underlying algorithms. In detail, self-calibration describes the estimation of intrinsic and extrinsic camera parameters from observed point correspondences in an image sequence. Under affine projection, these parameters can be recovered via factorization [18] and the obtained results subsequently transformed to the euclidean space. The way this transformation is computed, is specified by the applied affine camera sub-model. Namely, the considered camera models are the orthographic [18], scaled orthographic [19], weak perspective and general affine camera [20]. The evaluation of the different self-calibration approaches and yielded reconstruction results allows to identify the most robust and accurate method for the available setup. For this purpose, the computed point clouds are directly compared to confocal laser scanning microscope (CLSM) data that represents a fully metric measurement. By measuring and reconstructing a reference geometry the yielded accuracies are first assessed.

The outlined reconstruction routine for SEM images has the advantage that it is principally not dependant on a special tilt motion, a previous full calibration or a large amount of images. By processing short image sequences, the accurate factorization approach can be applied that uses point correspondences visible in all images of a sequence to compute the parameters of the affine camera. The presented registration scheme allows to combine several reconstructions from largely altered views to overcome the limitations of short image sequences and reduce the overall amount of necessary images. To obtain meaningful representations of the imaged surfaces, dense correspondences are determined for rectified stereo-pairs. Therefore, existing rectification approaches are adapted and extended to solve this problem for the affine camera in an improved way so that a dense matching algorithm can be subsequently applied. With the camera parameters and determined dense correspondences a point cloud is finally triangulated. A metric result is obtained by considering the pixel scale of the image related to the stage’s untilted initial position. For the evaluation, several specimens are reconstructed from images acquired at varying magnifications ranging from 60 up to 400. The evaluations in this magnification range allow to specify the findings of previous studies [8,15,16] and what reconstruction accuracies can be obtained using affine cameras to model the SEM in the context of self-calibration.

The outline of this paper is arranged in the following manner. In Section 2, the affine camera models are introduced and the methods applied in the reconstruction routine are elucidated along with the final registration step. The obtained results for varying specimen are presented in Section 3 and further discussed in Section 4. Finally, Section 5 draws the conclusions of the presented work.

## 2. Methods

**Figure 1:**
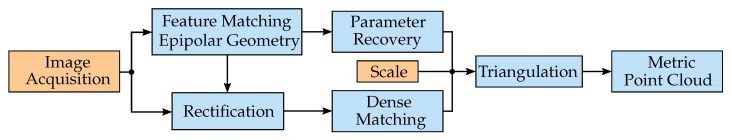
Schematic of the reconstruction routine’s processing steps. Data that is given to the pipeline is coloured in light red, the algorithmic processing of the data is depicted in blue.

In this section, the methods of the proposed reconstruction routine, illustrated in Figure 1, are outlined. At first, the affine camera along its related sub-models is introduced and the applicability to the SEM is theoretically justified. In succession, the acquisition of image sequences based on the equivalence of camera and object motion is described for the special case of the SEM. Then, the robust matching of image correspondences is elucidated as well as the information that can be extracted from the affine epipolar geometry. Subsequently, the self-calibration via factorization is explained, followed by specifying the metric constraints for the different affine camera sub-models and pointing out how to solve them for the desired upgrading transformations to euclidean space. To perform a row-wise dense stereo matching, the rectification problem for images under an affine projection is addressed. Finally, the dense matching procedure is described along with the structure computation via triangulation.

### 2.1. Affine Camera Model

**Figure 2:**
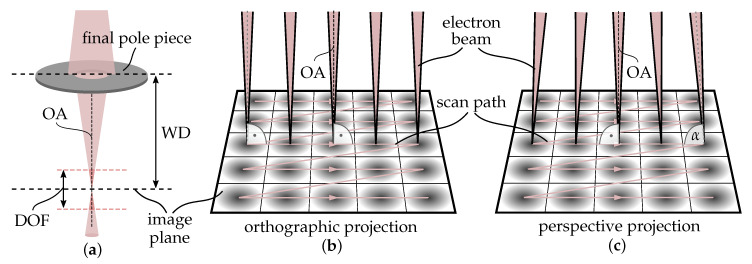
(**a**) Illustration of the working distance (WD) of a scanning electron microscope (SEM), its depth of field (DOF) and the optical axis (OA). (**b**) Ideal orthographic projection of the electron beams. (**c**) Perspective effects diminish for a small field of view as well as large ratios between the WD and depth variation of the scanned area. The smaller the field of view, the closer the angle α gets to perpendicularity.

In contrast to the common perspective camera, the camera center of the affine camera lies at infinity resulting in parallel projection rays. This has the effect that images are not altered when an object is translated parallel to the optical axis which means that the projection of an ideal affine camera is not scaled by the individual depth of the object points. These projection properties cause ambiguities in the case of motion recovery that can only be uniquely solved when three or more views of a scene are available [17,21]. The theory of these effects was deeply studied in the early works of computer vision. Nowadays, it finds its application in several disciplines like modelling imaging systems that use telecentric lenses [22], pushbroom cameras that provide satellite images [23] or in this scenario the SEM’s imaging system. Although all these systems do not fully comply with an ideal affine projection, its application is especially suitable when the depth relief of the scene is small compared to its average depth and when the distance of a considered point to the optical axis is small. The fact that the SEM’s working distance is usually much larger than the distances defined by the dimensions of the imaged scene causes the electron beam to be nearly parallel on every position of the raster scan pattern. In Figure 2b, the electron beams for an ideal orthographic projection and in Figure 2c, for a projection with remaining perspective effects are schematically illustrated. For a small field of view as well as high ratios between the working distance and depth variation of the scanned area, these perspective effects diminish.

The working distance of a SEM is defined as the distance between the pole piece of the final lens to the sample when its image is in focus as illustrated in Figure 2a. In “worst-case” scenarios, considering the example of investigating a sphere with a 1 mm diameter using a working distance of 10 mm, this ratio is 10. In the fields of computer vision, this number is treated as a satisfying bound to apply the affine projection model to common pinhole cameras since a long time [24]. With rising magnification the dimensions of the scene become smaller and the application of the model gets more precise.

In general, the affine camera $P_{A}$ maps a 3D world point $X={\left(X,Y,Z,1\right)}^{T}$ onto a 2D image point $x={\left(x,y,1\right)}^{T}$ in the form $x=P_{A}X$. $P_{A}$ is defined as

(1) $$P_{A}=\left(\begin{array}{cc} K_{2\times 2} & 0_{2\times 1}\\ 0_{1\times 2} & 1 \end{array}\right)\left(\begin{array}{cc} R_{2\times 3} & t_{2\times 1}\\ 0_{1\times 3} & 1 \end{array}\right)=\left(\begin{array}{ccc} c\cdot k & 0 & 0\\ s & k & 0\\ 0 & 0 & 1 \end{array}\right)\left(\begin{array}{cccc} r_{11} & r_{12} & r_{13} & t_{1}\\ r_{21} & r_{22} & r_{23} & t_{2}\\ 0 & 0 & 0 & 1 \end{array}\right),$$

where the 2×2 matrix K contains the intrinsic parameters and R describes the first two rows of a rotation matrix that defines the extrinsic parameters along with the translation vector t. The intrinsic parameters for the general affine camera model are the scaling factor *k*, an additional one-axial scaling factor *c* that allows an anisotropic scaling of the two image axes and the skew factor *s* that describes the angle between the images’ coordinate system axes being zero for square pixels. Distortions of the primary electron beam could lead to values for *s* different from zero resulting in transforming the images’ pixels to parallelograms. The scaling factor *k* takes magnification variations between images into account. Compared to the perspective camera, where every point is scaled by its individual depth, the average depth of the scene is used for an approximation [17].

Based on the calibration results of Cui et al. [15], it is expected that the parameter *c* is close to one and *s* is close to zero but with the addition that these two parameters can vary for different SEMs being affected by the construction of the electron column and the individually performed scanning process. The weak-perspective camera $P_{\mathrm{WP}}$ fixes the skew factor to a constant value of s=0 and uses the scaling factors *k* and *c* as variables. If only a uniform scaling of the images is considered such that c=1, the camera is denoted as a scaled orthographic camera $P_{\mathrm{SC}}$. Finally, the orthographic camera $P_{\mathrm{OR}}$ models the projection as purely orthographic, letting K become an identity matrix resulting in

(2) $$P_{\mathrm{OR}}=\left(\begin{array}{ccc} 1 & 0 & 0\\ 0 & 1 & 0\\ 0 & 0 & 1 \end{array}\right)\left(\begin{array}{cccc} r_{11} & r_{12} & r_{13} & t_{1}\\ r_{21} & r_{22} & r_{23} & t_{2}\\ 0 & 0 & 0 & 1 \end{array}\right).$$

### 2.2. Stereo Image Acquisition

**Figure 3:**
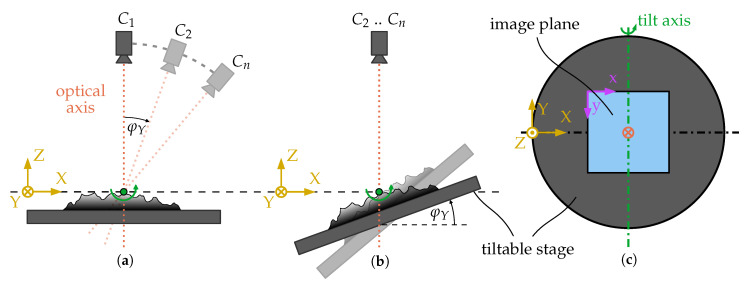
Equivalence of camera (**a**) and object motion (**b**) for a rotation angle $\varphi_{Y}$; (**b**) Eucentric tilting process with fixed eucentric point (green); (**c**) Adjustment of the scan rotation leading to the parallelism of the tilt axis and the image’s *y*-axis.

To capture images from different perspectives, the investigated sample is mounted on a tiltable stage. Due to the equivalence of camera and object motion, images are generated as if a camera is rotated around the object as illustrated in Figure 3a,b. Under ideal circumstances, the scan rotation, which is equivalent to the rotation of the image plane around the optical axis, and the stage’s height are adjusted so that the tilt axis lies directly in the image plane and parallel to the image’s *y*-axis what is depicted in Figure 3c. This setting results in a so-called eucentric tilting that causes a pure horizontal parallax between the corresponding points of a stereo-pair image while keeping the observed center point in focus. Under real conditions, the perfect adjustment of the tilt axis is not possible which causes additional small rotations around the *X*- and *Z*-axis as well as slight translations. The translational motion components are readjusted using the three axes of the stage to avoid a defocusing and alteration of the centered point. All other imaging settings are kept constant throughout the image sequences so that possible image changing effects are restricted to the varying position of the specimen along the *Z*-axis caused by imperfect stage readjustments. Under the presence of perspective effects, this translation can have a small magnifying or shrinking impact onto the images.

### 2.3. Feature Matching and Epipolar Geometry

To find corresponding points in the images of a sequence, the sequence is first handled as a string of stereo-pair images so that a feature detection and matching can be executed between the neighbouring images. A good and robust choice for SEM images is the well-known scale invariant feature transform (SIFT) that uses the difference of gaussians to detect suitable keypoints in the images [25,26]. The matching between keypoints in stereo images is carried out using a descriptor that describes the pixel surroundings of a keypoint. In the case of SIFT, the descriptor provides robustness towards slightly varying illuminations that can occur when the view on a specimen changes. To further improve the performance of the matching procedure, instead of the typical euclidean distance, the Hellinger kernel is used to measure the similarity between the SIFT descriptors which are basically histograms. It is known, that the Hellinger kernel yields a superior performance in the field of histogram comparison than the euclidean distance resulting in a higher amount of matches and less outliers [27].

To consider only high qualitative matches, for every feature point the ratio of the two best similarity scores is compared to a threshold and the match is discarded if the threshold is exceeded. Nevertheless, this procedure is still error-prone and more selective and robust ways to eliminate outliers are needed. Therefore, the properties of the tilting motion described in Section 2.2 are used to define additional constraints on inlying matches. For images acquired by this tilting method, it is possible to consider all matches that undergo a translation along the *y*-axis that is higher than a defined value as outliers. In a similar manner, a maximum distance for the *x*-translation can be defined. Both values have to be adjusted depending on the individual specimen and tilting process. Especially in tough matching scenarios for images captured from strongly varying perspectives, as used for the registration process described in Section 2.7, this filtering allows to prepare a correspondence set with a much better inlier ratio. This has a beneficial impact on the subsequent application of the robust estimation schemes.

To finally detect the still present noisy and faulty data in this set, the affine epipolar geometry of neighbouring stereo-pairs is robustly estimated to identify the correspondences that fit the determined model. In general, the epipolar geometry of two views is described by a 3×3 matrix, termed the fundamental matrix F, that maps points in the first image onto lines in the second one. For the affine camera, due to the parallel projection rays, these lines are also parallel to each other resulting in the affine fundamental matrix $F_{A}$ with an upper left 2×2 sub-matrix filled with zeroes written as

(3) $$F_{A}=\left(\begin{array}{ccc} 0 & 0 & a\\ 0 & 0 & b\\ c & d & e \end{array}\right).$$

**Figure 4:**
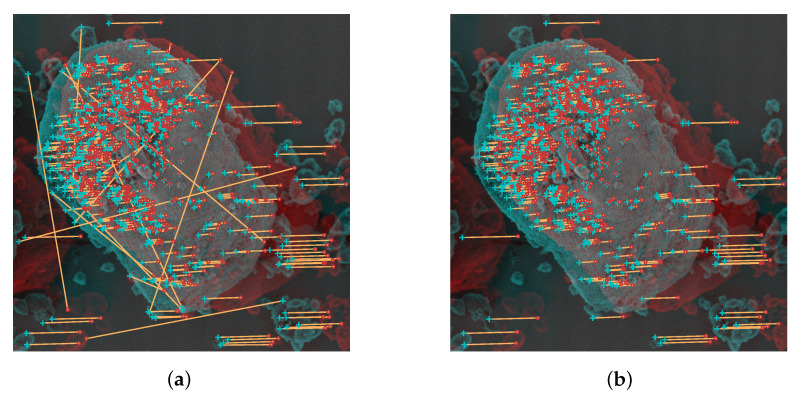
Exemplary anaglyph of a diesel exhaust particle (DEP) agglomerate ($\Delta \varphi_{Y}=15^{\circ }$); (**a**) Initial descriptor-based matching (Th=0.75), 711 matches; (**b**) Motion constraints ($\Delta y_{\max}=25$ px, $\Delta x_{\max}=200$ px) and Maximum Likelihood Estimation Sample Consensus (MLESAC) with σ=1 px, 617 matches.

This matrix can be estimated from a minimum amount of four point correspondences using the Affine Gold Standard Algorithm [28] which yields the Maximum Likelihood estimate of $F_{A}$ under the assumption that the noise in the point correspondences that are finally considered as inliers can be described by an isotropic and homogeneous Gaussian distribution. For low acceleration voltages of the primary electron beam and an EVT detector, Joy et al. [29] demonstrated that the noise in the secondary electron signal can be described by a Gaussian distribution as long as the set dwell time, the time in that electrons are detected to form the signal that is processed to the greyscale value of a certain pixel, is high enough, which is generally the case. Based on this approach, the Maximum Likelihood Estimation Sample Consensus (MLESAC) is used to obtain a robust estimate of $F_{A}$ and an outlier-free set of point correspondences for every stereo-pair [30,31]. The basic sampling procedure of a random sample consensus (RANSAC) uses point sets of the minimum configuration to compute the desired model [32]. MLESAC adopts this general sampling procedure but maximizes the likelihood of the solution instead of looking for the point set that maximizes the amount of inliers. Instead of maximizing the likelihood, the problem can be turned into minimizing the negative log likelihood and accordingly a robust error function $C_{\mathrm{ML}}$ for the point data can be defined in the form

(4) $$C_{\mathrm{ML}}=-\sum_{i}^{}\log\left(\gamma \frac{1}{\sqrt{2\pi \sigma^{2}}}\;\;\exp\left(\frac{-d_{e,i}^{2}}{2\sigma^{2}}\right)+\left(1-\gamma \right)\frac{1}{v}\right),$$

where the first term represents the Gaussian error distribution for inliers and the second term represents the uniformly distributed errors for outliers. γ denotes the mixing parameter which can be computed using the expectation minimization algorithm [30]. *v* is a constant that is defined by the before used motion constraint and the resulting limited search range for inliers in the image. As an appropriate residual $d_{e}$ for the MLE error in the affine projection scenario, the symmetrical epipolar distance is used which can be directly computed from the affine fundamental matrix and the coordinates of the correspondences [28]. It describes the squared orthogonal distance from a mapped epipolar line to its corresponding feature, summing up the values for both images. For the Gaussian distribution, the standard deviation is empirically set to σ=1 px. The related threshold *T* that is used to divide between in- and outliers for the finally obtained model is set to T=1.96σ, so that only 5% of inlying Gaussian errors are rejected. From all inlying point correspondences $F_{A}$ is reestimated and new inlying correspondences are determined. This guided matching (GM) is repeated iteratively until the number of correspondences is stable. In Figure 4, the initially matched point correspondences and finally obtained robust inlying matches are illustrated for an examplary stereo-pair. By using an anaglyphic visualization of the stereo images, the majorly horizontal translations of the inlying correspondences between the images caused by the eucentric tilting can be directly recognized.

On this basis, the ratio of the stereo-pair’s scaling factors $k_{1}$ and $k_{2}$ along with the in-plane rotation angle $\varphi_{Z}$ around the optical axis can be directly computed from the entries of the affine fundamental matrix according to Shapiro [17]. The orientations of the projected epipolar lines are described by *a* and *b* for the second image and by *c* and *d* for the first one so that the angles $\varphi_{Z_{1}}$ and $\varphi_{Z_{2}}$ can be computed in the form

(5) $$tan\left(\varphi_{Z_{1}}\right)=\frac{c}{d}\;\mathrm{and}\;tan\left(\varphi_{Z_{2}}\right)=\frac{a}{b}.$$

The relative scaling factor $k_{s}$, describing the scale change between the first view with $k_{1}=1$ and the second view with $k_{2}$, can be determined by the ratio of the sum of squared parameters a,b and c,d in the form

(6) $$k_{s}^{2}={\left(\frac{k_{2}}{k_{1}}\right)}^{2}=\frac{c^{2}+d^{2}}{a^{2}+b^{2}}.$$

**Figure 5:**
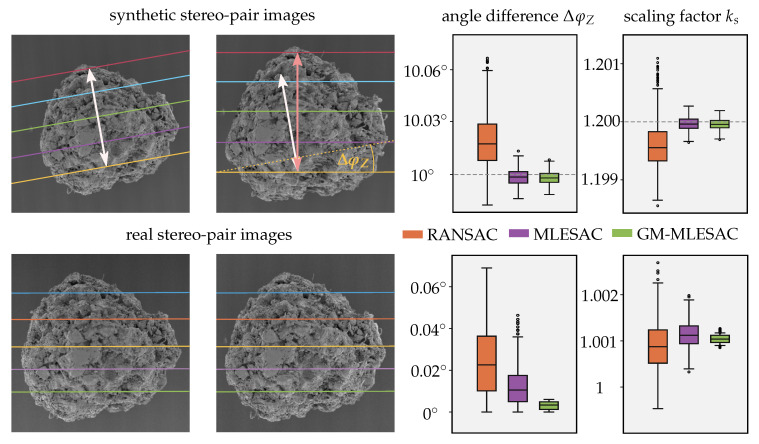
Synthetic and real stereo-pair of a gravel particle. Computation of the relative scaling factor $k_{s}$ and the angle difference $\Delta \varphi_{Z}=\varphi_{Z,1}-\varphi_{Z,2}$ using 1000 runs of the robust estimation schemes with 500 subsamples each. Rotation angles for the synthetic pair were set to $\Delta \varphi_{X}=0^{\circ },\Delta \varphi_{Y}=10^{\circ },\Delta \varphi_{Z}=10^{\circ }$ and the relative scaling factor to $k_{s}=1.2$. For the real images an eucentric tilting was used with $\Delta \varphi_{Y}=10^{\circ }$.

This factor directly corresponds to the ratio of the distances between the epipolar lines in the two images. In Figure 5 the different scaling of the distances between the epipolar lines is illustrated for a synthetic and a real stereo-pair of a gravel particle. The affine epipolar geometry was used to compute the angle difference of the two epipolar line slopes $\Delta \varphi_{Z}$ and the relative scaling factor $k_{s}$. The performance of the robust estimation schemes is illustrated and shows that MLESAC produces less scattered data based on the results of 1000 runs in comparison to RANSAC. The obtained results for the synthetic images are close to the true values. For the real images the ground truth is unknown because the adjustment of the scan rotation and the tilting of the stage are afflicted with uncertainties. The GM and computation of $F_{A}$ over all inliers allows to tighten up the result’s distribution. Probably due to the noise and the higher corruption of minimal configuration sets, this effect is more pronounced for the real images. The alteration of the scaling factor for the eucentric tilt is very small and lies only at about 0.1%. This scaling change between different views of an image sequence is caused by the alterations of the specimen’s position along the *Z*-axis due to the tilting process. Nevertheless, these very small deviations to a constant value of 1 could still be caused by the noise in the point correspondences. In the following sections, it will be further evaluated if the consideration of scaling changes in this reconstruction routine is beneficial and how it varies in its magnitude for other image pairs and sequences.

### 2.4. Parameter Recovery

To recover the intrinsic and extrinsic parameters of the affine camera for the acquired image sequence, the correspondences that can be observed in all of its images must be identified. Under affine imaging conditions, Tomasi and Kanade [18] demonstrated that the camera’s motion can be solely described by rotations when the measured point correspondences are centered around their local coordinate system. Therefore, the centered feature coordinates are written as a 2m×n measurement matrix $\tilde{W}$, where *m* denotes the number of point correspondences and *n* the number of image frames. It is desired to estimate the camera’s motion ${\hat{M}}_{A}$ and the 3D location of the sparse feature points ${\hat{X}}_{A}$ in affine space such that the distance between the estimated image points $\hat{x}=M_{A}X_{A}$ and detected image points x in the form $\sum_{f=1,j=1}^{n,m}\left|\right|x_{j}^{f}-{\hat{M}}_{A}^{f}{\hat{X}}_{A,j}{\left|\right|}^{2}$ is minimized. Writing the point correspondences in matrix form allows the creation of the term $\left|\right|\tilde{W}-{\hat{M}}_{A}{\hat{X}}_{A}{\left|\right|}^{2}$. Its minimization can be optimally performed using the following singular value decomposition $\tilde{W}=U\Sigma V^{T}$ so that the estimates ${\hat{M}}_{A}=U\Sigma^{\frac{1}{2}}$ and ${\hat{X}}_{A}=\Sigma^{\frac{1}{2}}V$ are obtained. These results are affine reconstructions and differ from the Euclidean ones up to an unknown affine transformation in form of a 3×3 matrix Q that upgrades the obtained affine structure and motion to the euclidean space. In order to determine the desired transformation Q, specific constraints are used based on the parametrization of the underlying affine camera model.

For the orthographic camera, it is taken advantage of the fact that for every frame *f*, the entries of the final motion matrix must represent a rotation matrix. Therefore its rows $i_{f}$ and $j_{f}$ must be unit vectors and orthogonal to each other, yielding the equation system

(7) $$i_{f}^{T}QQ^{T}i_{f}=1,\;j_{f}^{T}QQ^{T}j_{f}=1,\;i_{f}^{T}QQ^{T}j_{f}=0.$$

By defining $L={\mathrm{QQ}}^{T}$, the equation system can be solved for L according to [33]. Q can be subsequently determined from L using Cholesky or eigen decomposition that yield equivalent results. In the scenario of a non-positive definite matrix L, this necessary requirement is enforced by computing the nearest positive definite matrix to allow the decomposition for Q [34]. A non-positive definitiness of this matrix can be caused by too much noise in the point correspondences, remarkable perspective effects, insufficient rotational motion or an entirely planar sample shape [19]. With the transformation Q, the final results $\hat{M}={\hat{M}}_{A}Q$ and $\hat{X}=Q^{-1}{\hat{X}}_{A}$ are determined.

Regarding the scaled orthographic camera, the former orthogonality constraint should be satisfied as well. Though, the rows can be uniformly scaled for every frame relative to the scale of the first frame that is fixed to 1. Therefore, the equation system becomes according to Poelman and Kanade [19]

(8) $$r_{f}^{T}QQ^{T}s_{f}=0,\;r_{f}^{T}QQ^{T}r_{f}-s_{f}^{T}QQ^{T}s_{f}=0,\;r_{1}^{T}QQ^{T}r_{1}=1$$

with $r_{f}$ and $s_{f}$ being the rows of the final motion matrix. This matrix additionally contains the scaling factor for each row. Thus, the scaling factors for each row $k_{r,f}$ and $k_{s,f}$ as well as the rotation matrix rows $i_{f}$ and $j_{f}$ can be obtained by

(9) $$k_{r,f}=\left|\right|r_{f}||,\;k_{s,f}=\left|\right|s_{f}\left|\right|\;\mathrm{and}\;i_{f}=\frac{r_{f}}{k_{r,f}},\;j_{f}=\frac{s_{f}}{k_{r,f}}.$$

Due to noise, the scaling factors $k_{r,f}$ and $k_{s,f}$ will not be completely equal so that their mean is taken for every frame. The factorization based computation only differs from the before introduced method of Equation (7) using the epipolar geometry for a neglectable amount when the same set of point correspondences is used. In the following sections, the respective application of each method is specified.

For the weak-perspective camera, a cost function $C_{\mathrm{WP}}$ is formulated according to Quan [20] that allows the consideration of an anisotropic scaling of the image’s axes while maintaining the orthogonality constraint resulting in

(10) $$C_{\mathrm{WP}}=\sum_{f=1}^{m-1}{\left(\frac{r_{f}^{T}Q_{\rho }Q_{\rho }^{T}r_{f}}{s_{f}^{T}Q_{\rho }Q_{\rho }^{T}s_{f}}-\frac{r_{f+1}^{T}Q_{\rho }Q_{\rho }^{T}r_{f+1}}{s_{f+1}^{T}Q_{\rho }Q_{\rho }^{T}s_{f+1}}\right)}^{2}+\sum_{f=1}^{m}{\left(r_{f}^{T}Q_{\rho }Q_{\rho }^{T}s_{f}\right)}^{2}.$$

To solve this non-linear minimization problem while imposing positive-definitness on $Q_{\rho }$, the matrix is Cholesky parametrized and the famous Levenberg-Marquardt algorithm is applied. As a starting point, the unity matrix is used. Quan proved that the desired upgrading transformation Q can be determined up to an unknown rotation $R_{r}$ for the given $Q_{\rho }$. To fix $R_{r}$ and obtain Q with regards to selecting the first frame as a reference view, ${\hat{M}}_{A}^{1}Q_{\rho }$ is decomposed into the orthonormal matrix $R_{1}$ and the lower triangular matrix $K_{1}$ allowing to compute $Q=Q_{\rho }R_{1}^{T}$. The intrinsic parameters $K_{f}$ for each view can be determined using the related frames’ motion matrices ${\hat{M}}_{A}^{f}$ so that individual scaling factors $k_{f}$ are obtained.

To additionally consider skew, Quan exchanged the orthogonality constraint of Equation (10) with a term that comprises a constant skew value over all views which results in

(11) $$C_{A}=\sum_{f=1}^{m-1}{\left(\frac{r_{f}^{T}Q_{\rho }Q_{\rho }^{T}r_{f}}{s_{f}^{T}Q_{\rho }Q_{\rho }^{T}s_{f}}-\frac{r_{f+1}^{T}Q_{\rho }Q_{\rho }^{T}r_{f+1}}{s_{f+1}^{T}Q_{\rho }Q_{\rho }^{T}s_{f+1}}\right)}^{2}+{\left(\frac{r_{f}^{T}Q_{\rho }Q_{\rho }^{T}s_{f}}{s_{f}^{T}Q_{\rho }Q_{\rho }^{T}s_{f}}-\frac{r_{f+1}^{T}Q_{\rho }Q_{\rho }^{T}s_{f+1}}{s_{f+1}^{T}Q_{\rho }Q_{\rho }^{T}s_{f+1}}\right)}^{2}.$$

This cost functions can be minimized using at least four images and the intrinsic parameters are determined in the same way as for the weak-perspective camera. The axial scaling factor *c* and the skew factor *s*, which are constrained to be equal over all views, are in both cases computed from the matrices $K_{f}$ as a mean over all frames.

### 2.5. Rectification

The search for dense correspondences between two stereo-pair images is a computational expensive task. Therefore, the problem is usually reduced to a 1D search along conjugate epipolar lines. The direct computation of these skewed pixel paths in the images is cumbersome and can be simplified by transforming the images so that corresponding points are located in the same pixel rows of both images. After the rectification, the new pixel coordinates are given as floating point numbers. Therefore, the new images are resampled using bilinear interpolation.

**Figure 6:**
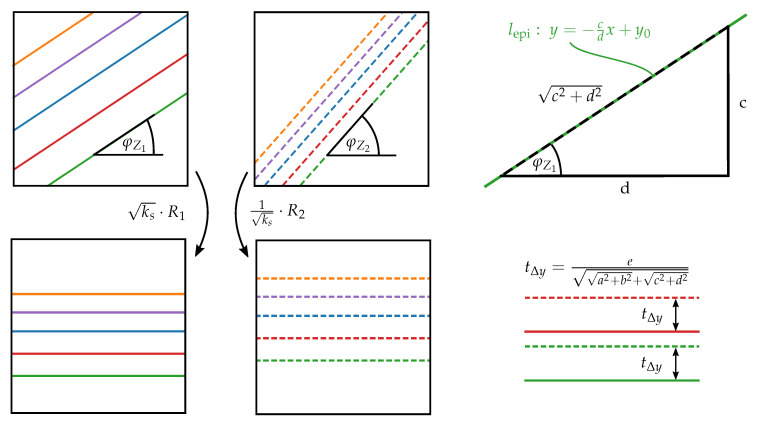
Unrectified epipolar lines for an affine projection with the slope angles $\varphi_{Z_{1}}$ and $\varphi_{Z_{2}}$. Applying the rotations $R_{1}$, $R_{2}$ and the scaling adjustment results in horizontal and evenly spaced epipolar lines. Finally, the translational offset $t_{\Delta y}$ can be compensated. In the top right, the definition of an epipolar line $l_{\mathrm{epi}}$ is given and the triangle that allows to define the rotation matrices of Equation (12).

Due to the special form of the affine fundamental matrix given in Equation (3), the epipolar lines in every image are parallel to each other. For images taken with an ideal orthographic camera, they are also equally spaced, which allows to solve the rectification problem by adjusting the slope of the epipolar lines with a rotation around the optical axis and further align the *y*-coordinate displacement with a translation. This approach was used in [14] to rectify SEM images resulting in useful epipolar line alignments. The necessary rotation matrices can be defined by determining the slope of the epipolar lines for every image given in Equation (5) or directly using the unit vectors of the epipolar lines’ orientations, illustrated in Figure 6, that directly correspond to the entries of the desired rotation matrix. For the first image, this matrix is given as

(12) $$R_{1}=\left(\begin{array}{ccc} \cos\;\varphi_{Z_{1}} & -\sin\;\varphi_{Z_{1}} & 0\\ \sin\;\varphi_{Z_{1}} & \cos\;\varphi_{Z_{1}} & 0\\ 0 & 0 & 1 \end{array}\right)=\frac{1}{\sqrt{c^{2}+d^{2}}}\left(\begin{array}{ccc} d & -c & 0\\ c & d & 0\\ 0 & 0 & \sqrt{c^{2}+d^{2}} \end{array}\right)$$

and vice versa $R_{2}$ for the second one substituting the parameters *c* and *d* with −a and −b or rather $\varphi_{Z_{1}}$ with $-\varphi_{Z_{2}}$. The missing translation that compensates the *y*-coordinates’ offset can be easily found by computing the mean *y*-distance between corresponding feature points in the rotated images.

To consider the scaling factors $k_{f}$ of the different views and take advantage of all information provided by a stereo-pair image under affine projection which allows to obtain an improved rectification result, the computation of a similarity transform is introduced based on the theoretical findings of Shapiro [17] and Shimshoni [13]. For this purpose, one image has to be adapted to fully match the other or the scale adjustment has to be equally split onto both images. In the scenario of rectification, the latter approach should be used because it is desired to keep the distortions of a single image at a minimum changing the original image information as little as possible. Therefore, the relative scaling factor between the views $k_{s}$ is split up and applied to the transformations in a magnifying and shrinking manner. $k_{s}$ can be computed according to Equation (6) for a fixed $k_{1}=1$. Therefore, $k_{s}=\frac{k_{2}}{k_{1}}$ can be solved for the new adjustment factors under the condition that $k_{1}=\frac{1}{k_{2}}$ which results in the equally split scaling. The obtained adjustment factors are $p_{1}=\sqrt{k_{s}}$ and $p_{2}=\frac{1}{\sqrt{k_{s}}}$ which are subsequently applied to the images’ rotation matrices of Equation (12). The resulting similarity transformations yield equally spaced and purely horizontal epipolar lines as illustrated in Figure 6. The still present *y*-coordinate displacement can be computed from the so far ignored parameter *e* that is directly related to the orthogonal component of translation between the epipolar lines of the two images. To represent this relative translational difference between the scale adjusted images, *e* has to be scaled accordingly. Combining the prefactors of the rotation matrices with the scale adjusting factors, written as

(13) $$p_{1}\cdot \frac{1}{\sqrt{c^{2}+d^{2}}}=p_{2}\cdot \frac{1}{\sqrt{a^{2}+b^{2}}}=\frac{1}{\sqrt{\sqrt{a^{2}+b^{2}}+\sqrt{c^{2}+d^{2}}}},$$

it emerges that the matrix entries *a*, *b*, *c* and *d* of the similarity transforms for both images are scaled by the same factor in the case of an equally distributed scale adjustment. Therefore, *e* has to be multiplied as well with this factor to represent the relative translational difference along the *y*-axis in the scale adjusted and rotated images so that the missing translation can be defined as

(14) $$t_{\Delta y}=\frac{e}{\sqrt{\sqrt{a^{2}+b^{2}}+\sqrt{c^{2}+d^{2}}}}.$$

This translational component is applied to the first image. The final rectifying similarity transforms can subsequently written as

(15) $$S_{1}=\sqrt{k_{s}}\cdot R_{1}+\left(\begin{array}{c} 0\\ t_{\Delta y}\\ 0 \end{array}\right)\;\mathrm{and}\;S_{2}=\frac{1}{\sqrt{k_{s}}}\cdot R_{2}.$$

**Figure 7:**
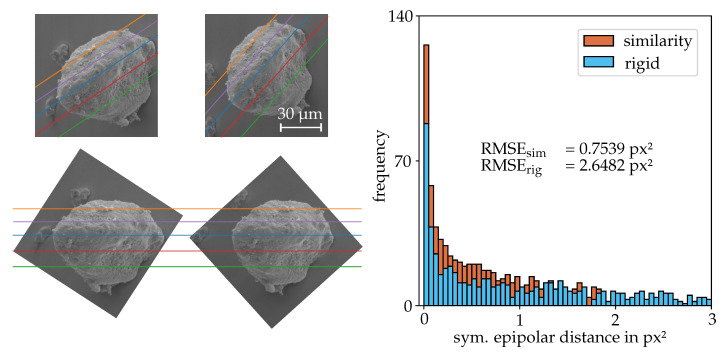
(left) Unrectified and rectified stereo-pair using the similarity transform for the images of a DEP agglomerate (DEP II). (right) Post rectification errors computed for the two different methods in terms of the symmetrical epipolar distances using $F_{R}$ for 463 matches.

It is further possible to minimize the images’ horizontal displacement which can be useful if the search range for the dense matching algorithm is otherwise very high. According to Hartley [28,35], the transformed point correspondences in the rectified images $x_{R,i}=\left(x_{R,i},y_{R,i},1\right)$ and $x_{R,i}^{\prime }=\left(x_{R,i}^{\prime },y_{R,i}^{\prime },1\right)$ can be used to minimize the quantity

(16) $$\sum_{i=1}^{m}\left(x_{R,i}\cdot k_{x}+s_{x}\cdot y_{R,i}+t_{x}-x_{i}^{\prime }\right)$$

in a least squares manner providing the scaling $k_{x}$, the translation $t_{\Delta x}$ and the skew $s_{x}$ that minimize the distances between the point correspondences along the *x*-axis. For the disparity of the SEM stereo-pairs acquired with a center point readjustment, it is sufficient to apply only the translational component $t_{\Delta x}$ to the first rectified image avoiding additional image distortions that would occur if the *x*-axis scaling and skew $s_{x}$ were applied to the images. In Figure 7 and Table 1, several examples of the rectification results are given for varying tilt motions. As an evaluation criterion, the symmetrical epipolar distances are computed for the point correspondences in the rectified images using the fundamental matrix $F_{R}$ of a perfectly rectified stereo-pair which takes the special form

(17) $$F_{R}=\left(\begin{array}{ccc} 0 & 0 & 0\\ 0 & 0 & -1\\ 0 & 1 & 0 \end{array}\right).$$

The similarity transform outperforms the rigid one, especially for stronger view alterations. The more precise the eucentric tilting is carried out, the better the initial rectified state of the images. In these scenarios, the results of both algorithms get very close. Nevertheless, for all considered stereo-pairs the final deviations to the rectified state are smaller using the similarity transform.

### 2.6. Dense Matching and Triangulation

**Figure 8:**
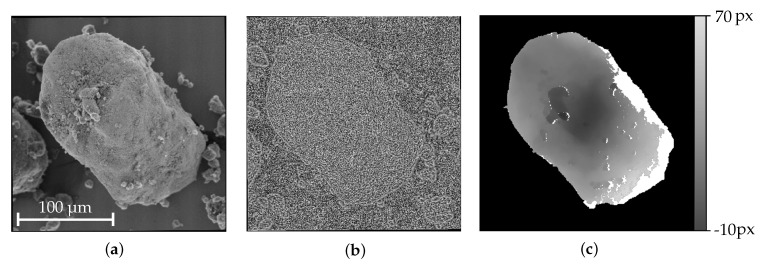
(**a**) Rectified image of a DEP agglomerate (DEP I); (**b**) Rank transformed image with a 9 × 9 window; (**c**) Disparity map illustrating the horizontal parallax between the images of a stereo-pair for a tilt angle of $\Delta \varphi_{Y}=10^{\circ }$ where black represents the background and white the area of the segmented particle. For the white parts, unreliable matches were discarded.

To determine a disparity map that contains the information about the horizontal parallax of corresponding pixels between the stereo-pair images, a dense matching algorithm is used. There are various approaches to solve the dense stereo matching problem. In this work, the famous Semi-Global Block Matching (SGBM) approach was chosen that provides a good trade-off between accuracy and computation time [36]. To compensate occuring radiometric distortions and improve the matching quality in the area of depth discontinuities, a rank filter is applied to the images that replaces the intensity values with the number of pixels which intensities are below the center pixel’s intensity for a certain neighbourhood [37]. The utilized SGBM implementation combines the pixelwise, local Birchfield-Tomasi Subpixel Metric [38] that computes the absolute distance between the extrema of linear interpolations of the corresponding pixels of interest with their neighbors and a global smoothness constraint that is approximated by computing eight symmetrical, one-dimensional pathwise optimizations for each pixel of the disparity map and summing their costs. Finally, for every pixel the disparity is chosen that has the lowest sum of aggregated costs. Low quality matches are discarded when the uniqueness defined as the ratio of the best two costs for different disparity values is too similar. Further outliers are identified by a left-right consistency check, switching the image of the stereo-pair in that is searched for the corresponding pixel. If the obtained difference of the two disparity values for every pixel surpasses a chosen threshold, none of the values is assigned to the related pixel. These post-processing steps ensure that occlusions and invalid matches do not falsify the final result. For optimal performance, the underlying thresholds are individually adjusted based on the imaged structures. In Figure 8, the SEM image, its rank transformed form and the obtained disparity map from the related stereo-pair are illustrated. To segment the specimen that functions as the region of interest for this reconstruction routine, a graph cuts based image segmentation approach [39] is used to only reconstruct the desired parts of the images.

The obtained disparity map allows to identify the corresponding pixels in the rectified images. Subsequently, the coordinates of these point matches are transformed back to the original image’s coordinate system applying the inverse rectifying transformations. In addition, the image points are centered at the origin of their local coordinate system to eliminate the translational vector describing the camera’s position. The metric scaling factor that was initially determined for the untilted stage setting using a multiscale checkerboard pattern, is applied to the point coordinates to transform the pixel values to metric ones. For images of SEMs, this factor is directly proportional to the selected magnification setting as long as the working distance and stage height are not altered. With the before recovered intrinsic and extrinsic parameters, the inhomogenious triangulation method [28] is used to solve for the 3D coordinates in the world coordinate system that coincides with the first frame’s coordinate system of the sequence.

### 2.7. Registration

As a final step, the results of several image sequences can be registered based on point correspondences between selected connection images. Large tilt angles between the sequences allow to capture larger parts of the specimen with a minimum amount of images. If only few correspondences can be found due to the large view alteration, the affine invariant feature algorithm Affine-SIFT [40] is used to increase their number. This method simulates a set of sample views of the original two images that are created by rotating every image around its *x*- and *y*-axis. To these transformed images the Root-SIFT method is applied and additionally found features are transformed back in the original image. Although the view changes are mainly caused by tilting the stage around the *y*-axis, also rotations around the *x*-axis are taken into consideration to further increase the number. The matching is carried out in the same manner as described in Section 2.3 with slightly adjusted parameters.

**Figure 9:**
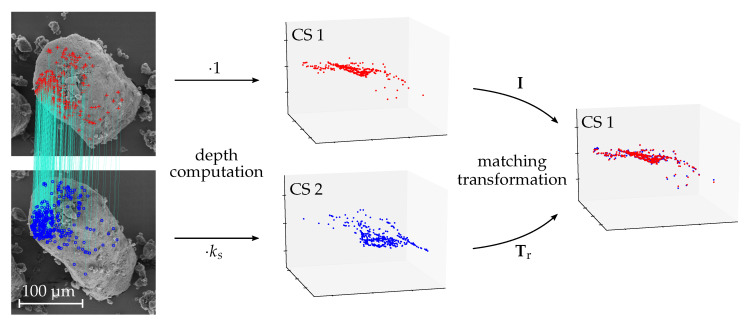
Computation of the rigid transformation $T_{r}$ for the initial alignment of the dense point clouds. The blue point set is scale adjusted and the computed transform maps it onto the red point set.

Subsequently, the depth, provided by the information of the related image sequence, is computed for the detected correspondences. Like illustrated in Figure 9, the obtained 3D point sets are further used to determine an initial rigid transformation $T_{r}$ that matches one point set onto the other according to Arun et al. [41]. This transformation is subsequently applied to the related dense point cloud for a rough alignment. In addition, the relative scaling factor $k_{s}$ between the connection images, computed according to Equation (6), is used for a scale adjustment. The cloud alignment is further refined using the Trimmed ICP algorithm [42] to minimize the C2C distances between the overlapping parts.

## 3. Results

**Figure 10:**
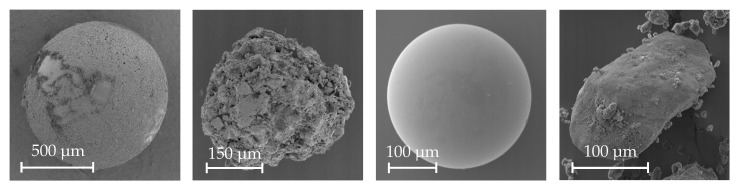
Images of the specimen that were used for the evaluation process. From left to right: Chalked Sphere (mag. 60, 2000 × 2000 px), Gravel Particle (mag. 150, 1000 × 1000 px), 300 µm Sphere (mag. 200, 2000 × 2000 px), DEP agglomerate (mag. 400, 2000 × 2000 px).

For the evaluation of the reconstruction routine, four different specimen were selected, illustrated in Figure 10, which represent varying surface structures and were captured at magnifications ranging from 60 to 400. In detail, these are two spheres measuring a diameter of 300 µm and 1000 µm, a gravel particle and a DEP agglomerate. All SEM images were acquired with a Zeiss DSM 940A (Carl Zeiss AG, Oberkochen, Germany) equipped with EVT detectors. Each image sequence consists of four images to allow the comparison of the four different ways to compute the upgrading transformation to Euclidean space. The tilt motions of the stage were carried out using $\Delta \varphi_{Y}\approx 5^{\circ }$ to generate the varying views onto the specimen. With the different four sets of provided parameters related to the four different methods, the same disparity map is used to triangulate a dense point cloud. The used magnifications for the images of the spheres were 60 and 200, for the gravel particle 150 and for the DEP agglomerate 400. Because the very low magnified images for the 1000 µm sphere do not depict its very fine surface details precisely, it was treated with a chalk spray to generate an optically cooperative surface that enables the identification of correspondences in sufficient number. The surface was further marked by a scratch to allow the unambiguous registration of the CLSM measurement and the SEM reconstructions. For the 300 µm reference sphere, this problem did not occur because the magnification is high enough to make the imperfections of the surface structure visible. Thus, features are provided naturally and no chalk was used. The given manufacturer specifications are the radius of 150 ± 2.5 µm and a sphericity of 0.625 µm. Since these values are known, a spherical fit is applied to the obtained point clouds in a LSQ sense. The radii of the fitted sphere and the deviations from the fitted geometry in normal direction are further compared with the given specifications and tolerances. For all other specimen, the reconstructions are registered to the CLSM measurement by an initial manual alignment and subsequent ICP refinement that is performed until the RMSE of the absolute euclidean distances between the nearest neighbouring points of the clouds, in the following denoted as cloud-to-cloud (C2C) distances, changes between two iterations less than $10^{-6}$ µm. After the alignment, the C2C distances between the compared SEM reconstructions and the CLSM measurements are used as an assessment criterion to estimate the accuracy of the obtained reconstruction results. The CLSM is a Keyence VK-X200K (Keyence, Osaka, Japan) which is specified to have a digital axial resolution of 0.1 nm with a measurement repeatability of 0.012 µm.

### 3.1. Reconstruction Evaluation

**Figure 11:**
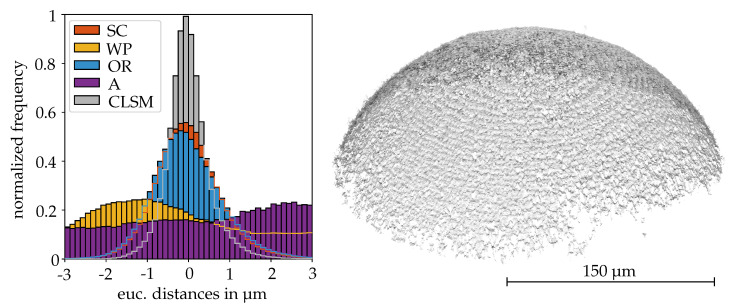
(left) Deviations of the reconstructed point clouds to a LSQ -sphere fit given in histogram form. For all results, a triangulation angle of $\Delta \varphi_{Y}=10^{\circ }$ was used. (right) Reconstructed point cloud of 1071344 points using the scaled orthographic factorization method.

First, the results for the 300 µm reference sphere are presented to classify the accuracy of the CLSM measurements and SEM reconstructions. In Figure 11, the deviations from the measurements to the spherical LSQ fit are illustrated in form of a histogram. It can be noticed that the data provided by the CLSM comes closest to the given manufacturer specifications and yields the smallest RMSE for the distances to the spherical LSQ fit which is given along with the RMSEs for the factorization methods, their recovered intrinsic parameters and the radii of the spherical LSQ fits $r_{\mathrm{LSQ}}$ in Table 2. For the reconstruction routine, the scaled orthographic factorization method obtained the most precise result, closely followed by the orthographic factorization method. The methods based on the non-linear minimization of a cost function produce distorted results which are mainly caused by the wrong estimation of the one-axial scaling factor *c* and the skew factor *s*. It is demonstrated in Table 3, that the adjustment of c=1 and s=0, after the computation of the extrinsic and intrinsic parameters using the weak perspective and general affine cost functions, results in a better overall reconstruction result although for the weak perspective camera, $r_{\mathrm{LSQ}}$ turns out to be too high. The radii for the CLSM measurement and the scaled orthographic factorization method lie in the tolerances of the manufacturer’s specifications. On the contrary, the orthographic factorization results in a $r_{\mathrm{LSQ}}$ that is too small. The recovered rotation angles are given exemplarily in Table 3 for the manually carried out stage motion with tilt angle changes of approximately $\Delta \varphi_{Y}\approx 5^{\circ }$ between the stage’s positions. In the following, the illustrations of the C2C distances are limited to the results that allow a meaningful registration with the CLSM measurements. An overview of the estimated parameters for the different methods is given in Table 4 so that the reasons for distorted reconstructions can be identified.

**Figure 12:**
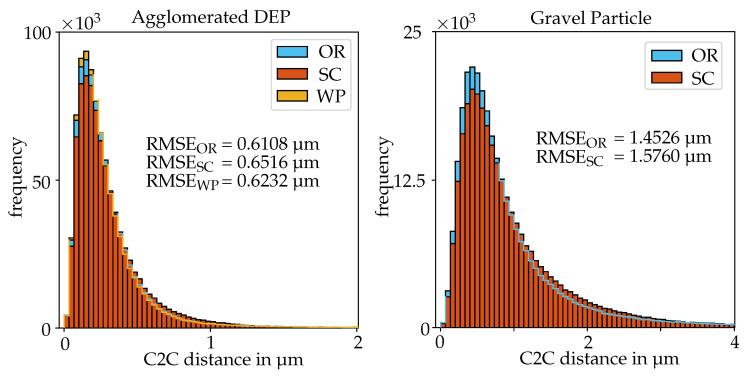
C2C distances between the aligned SEM reconstructions and confocal laser scanning microscope (CLSM) measurements for the respective factorization method regarding the sequences of the DEP agglomerate (1344742 points) and Gravel Particle (327997 points).

**Figure 13:**
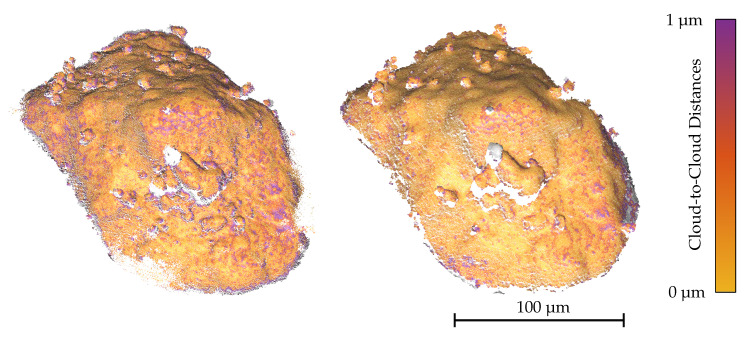
CLSM measurement (left, 969072 points) and the dense point cloud triangulated from a stereo-pair with a tilt angle of $\Delta \varphi_{Y}=10^{\circ }$ (right, 1344742 points) using the weak perspective factorization method. The cloud is coloured in the C2C distances between the two ICP registered point clouds. Unequal missing parts are coloured in white.

**Figure 14:**
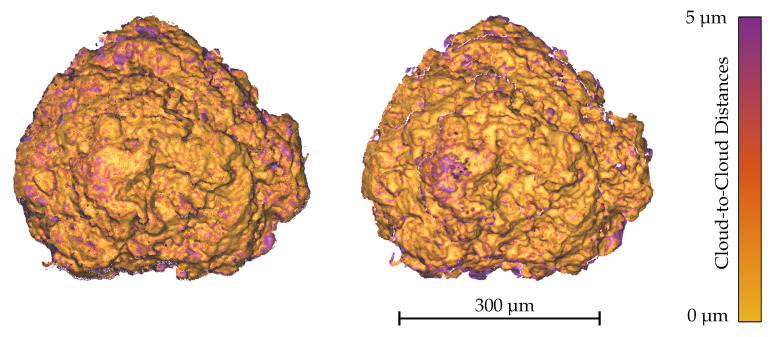
CLSM measurement (left, 1081582 points) and the dense point cloud triangulated with a triangulation angle of $\Delta \varphi_{Y}=5^{\circ }$ (right, 327997 points) using the orthographic factorization method for the image sequence Gravel Particle.

The C2C distances between the reconstructions of the DEP agglomerate and the CLSM measurement are illustrated in Figure 12. The results based on the orthographic, scaled orthographic and weak perspective camera models show similar deviations, however, the orthographic one generates the smallest RMSE for this image sequence. The recovered one axial scaling parameter *c* from the weak perspective cost function only deviates slightly from 1 with c=0.9975 in this scenario. In Figure 13, the dense point clouds for the weak perspective factorization and the CLSM measurement are depicted and compared to each other, demonstrating that the major parts of the surfaces coincide. The general affine cost function did not provide a meaningful result, yielding parameters that strongly deviate from the expected case. In Table 4, the parameters *c* and *s*, provided by the cost functions for the weak perspective and affine camera, are listed for all considered image sequences. The image sequence of the gravel particle, for which the results are depicted in Figure 12 and Figure 14, lets the methods based on the non-linear minimization scheme fail. In both scenarios, the triangulated point clouds are strongly distorted which is mainly caused by the wrongly estimated intrinsic parameters similar to the results of the 300 µm sphere sequence in Table 2. Therefore, meaningful registrations to the CLSM measurements are not possible. The other two methods based on linear LSQ yield comparable results to the DEP agglomerate sequence with the orthographic one providing a result with slightly less deviations to the CLSM measurement. Nevertheless, the overall RMSE for the Gravel Particle is higher. For one thing, this is probably caused by the demanding structure consisting of lots of steep edges that have an impact on the accuracy of the estimated disparity map and also causes the CLSM to measure less reliable in these parts. In addition, the magnification for the images of the gravel particle is lower and the object’s dimensions slightly larger. Therefore, the pixel constant, transforming the pixel values into metric ones and which also scales the resulting C2C distances, is as well naturally higher.

**Figure 15:**
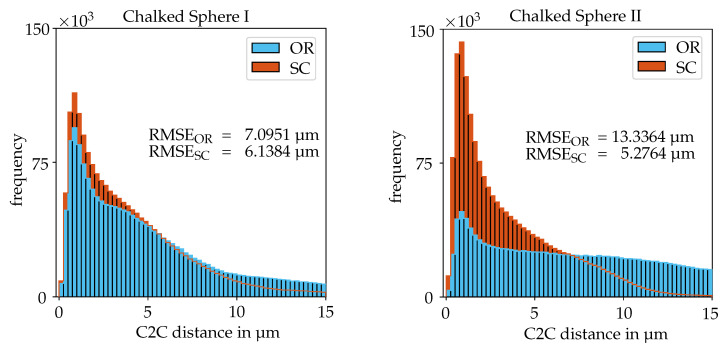
C2C distances between the aligned SEM reconstruction results and CLSM measurements for the respective factorization method regarding the image sequences of the Chalked Sphere (1855408 points).

The last specimen that was used for the evaluation is the chalked sphere. Its images were acquired at a very low magnification of 60. The deviations from the CLSM measurement are illustrated for two different image sequences as histograms in Figure 15. For the first sequence, the focus and *Z*-position of the stage were very carefully readjusted observing a feature on the surface at a higher magnification so that the possible translation of the focussed center point was reduced to a minimum. For the second one, the focussed center point translated slightly along the *Z*-axis so that its relative position to the focus plane changed due to the stage motion but kept the specimen still in a decent focus. For both sequences, the cost functions demonstrate once more that they do not provide robust results and determine intrinsic parameters that strongly differ to the expected orthographic scenario like shown in Table 4. The two linear methods, starting with the first sequence, yield accurate results, albeit the orthographic factorization provides a noticeably inferior RMSE. For the second sequence, this tendency is increased, generating a RMSE that is more than double the value of the scaled orthographic factorization method. The effect that leads to this outcome can be found in the zooming or rather shrinking caused by the translation of the specimen along the optical axis. This translation affects the images due to the still remaining perspective properties of the SEM’s imaging system at low magnifications. The scaled orthographic factorization method is able to overcome this difficulty more robustly while the orthographic factorization method only provides good results when the relative position of the specimen to the focus plane is not altered meaningfully during the sequence. Interestingly, the RMSE for the scaled orthographic factorization method decreases even further for the second image sequence. This result could be caused by the differently distributed feature correspondences due to the imperfectly focussed sphere tip. In Table 4, the scaling factors $k_{f}$ provided by the scaled orthographic factorization method are given along with the summed deviations to the orthographic scenario (k=1) to compare the different magnitudes for the varying image sequences. The translation of the specimen along the *Z*-axis results in an increase of the scaling factor $k_{4}$ compared to the reference image by approximately 1%. Although another set with less correspondences is found for the second sequence, the factors for the unchanged images barely vary. Taking all scaling factors of the different image sequences into consideration, it is noticeable that the overall deviation from a constant scaling factor decreases slightly for an increasing magnification. In general, for specimen with a smaller depth variation, a slightly different adjustment of the stage’s height over an image sequence is more unlikely to happen because small alterations of the specimen’s position along the *Z*-axis will quickly defocus all parts of it and the effect will be directly visible. In addition to the easier handling of the readjustments, the smaller dimensions of the scene and the smaller depth variations will decrease the remaining perspective effects.

In Table 5, the deviations between the expected tilt angles and the estimated tilt angles for the rotation around the *Y*-axis $\Delta \varphi_{Y,f}$ are given for all scenarios in that the orthogonal and scaled orthogonal factorization methods differed noticeably. Although, the exact angles are not known, a trend is noteworthy. It can be seen that in these scenarios not only the consideration of the scaling factor as an intrinsic parameter for the triangulation is necessary, but also that the recovered extrinsic parameters are directly affected by the underlying metric constraints that are used to solve for the upgrading transformation to Euclidean space. Thus, the selection of the factorization method should be made carefully according to the properties of the image sequence.

**Figure 16:**
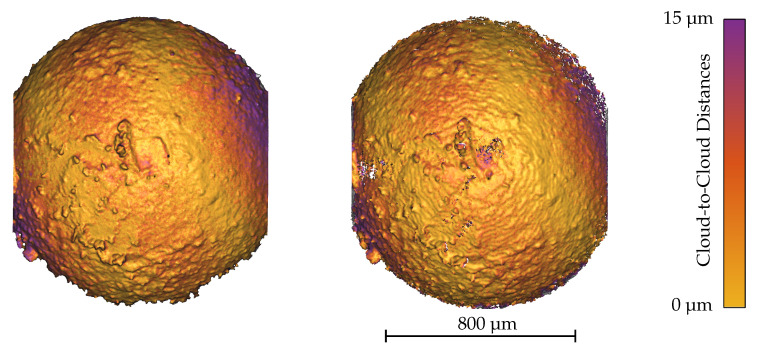
CLSM measurement (left, 2250786 points) and the dense point cloud triangulated with a triangulation angle of $\Delta \varphi_{Y}=5^{\circ }$ (right, 1855408 points) using the scaled orthographic factorization method for the image sequence Chalked Sphere I. Cuts are caused by the measurement field of the CLSM objective.

A final view on the details of the reconstructed surface of the Chalked Sphere in Figure 16 reveals the common limitations of photogrammetric approaches, solving the correspondence problem in smooth areas. The blank spots of the spherical surface cause some noise in the reconstructed point cloud and some minor surface details get lost. Nevertheless, major characteristics of the surface coincide in both measurements. The highest visible deviations are present on the right and left sides of the sphere. Adjusting the scale of the reconstructed sphere could not improve the result so that there is probably a small curvature mismatch. On the one hand, this could be caused by distortions of the scan pattern that can occur in the very low magnification range [43]. On the other hand, the presence of perspective effects may impair the factorization methods and lead to falsified reconstruction results.

### 3.2. Registered Point Clouds

**Figure 17:**
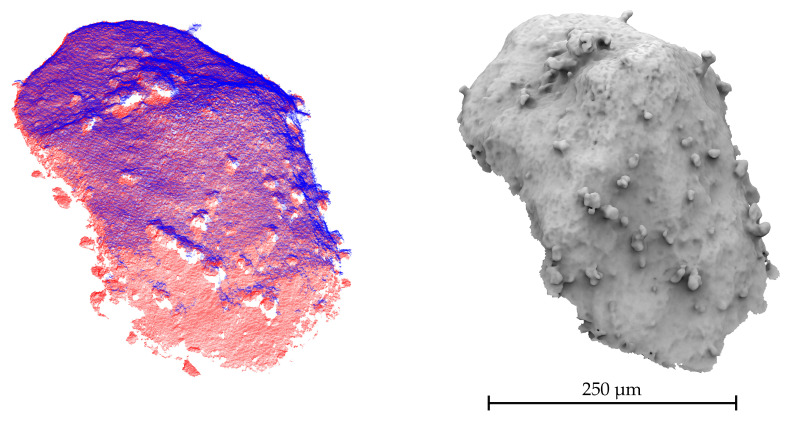
(**left**) Aligned dense point clouds, triangulated from the views ($-15^{\circ }$/$-10^{\circ }$) (red) and ($20^{\circ }$/$25^{\circ }$) (blue). (**right**) Poisson surface reconstruction created from the merged point clouds.

In Figure 17, the two registered and merged point clouds are illustrated. The images which function as the triangulation basis were selected for the stage’s rotation angles of $\varphi_{Y}$ = ($-15^{\circ }$, $-10^{\circ }$) and $\varphi_{Y}$ = ($20^{\circ }$, $25^{\circ }$) utilizing the full tilt motion of the available SEM stage. The additional views provide more data about the sloping surface parts so that the DEP agglomerate’s top surface can be completely reconstructed. As connection images, the views with the angle settings of $\varphi_{Y}=-10^{\circ }$ and $\varphi_{Y}=25^{\circ }$ were selected, in total a tilt angle difference of $\Delta \varphi_{Y}=35^{\circ }$. Due to the use of ASIFT, the number of valid correspondences between the connection images is increased from 177 to 437 so that the initial aligning transform can be robustly determined. Based on an estimated overlap of 80%, the Trimmed ICP algorithm is applied in the same manner as used for the comparison of the CLSM and SEM data. The final registration error for the 80% considered points of the dense point clouds was 0.7212 µm. This value is slightly reduced by adjusting the scale in the initial alignment with the computed $k_{s}$ for the connection images to 0.7205 µm. The aligned point clouds were further merged and a mesh was created using a Poisson Surface Reconstruction [44]. For each sequence, three images and the orthographic factorization method were used. Even though the impact of the scale adjustment is rather small in this scenario, it is more likely that an impact on the scaling between images with strong view alterations can appear. Due to the perspective change, different features of the surface have to be focussed and the depth profile will change. Furthermore, the image acquisition could still be extended to capture additional image sequences for differently rotated stage positions or larger view alterations.

## 4. Discussion

The presented results demonstrate that the outlined reconstruction routine based on the affine camera can be applied to SEM image sequences to obtain metric reconstruction results comparable to CLSM measurements. Nevertheless, the factorization algorithms used to recover the extrinsic and intrinsic parameters should be selected according to the magnification setting and the properties of the image sequence. Still, in the very low magnification scenario, the results seem to be affected by perspective or also distortive effects so that the reconstruction accuracy declines. For this magnification, the metric constraints of the scaled orthographic camera provide superior results. It was also demonstrated that alterations of the specimen’s position along the optical axis in the images of a sequence can be robustly handled. In higher magnification settings, the scaled orthographic factorization method seems to be slightly more vulnerable to noise compared to the basic orthographic approach. For carefully performed tiltings at magnifications higher than 150, the scaling factors of the individual images within a short sequence are very close to 1 and the impact of perspective effects was not noticeable so that sticking to the orthographic model seems reasonable. Nevertheless, the scaled orthographic factorization method is identified as the method with the most overall robustness and provides even for higher magnifications results similar to the orthographic one. The use of the cost functions for the weak perspective and general affine camera mostly resulted in distorted reconstructions. Even though these methods are generally more vulnerable to noise due to the iterative non-linear minimization procedure that is utilized, the true values of the one-axial scaling factor *c* and the skew factor *s* seem to be naturally very close to the assumptions made by the orthographic and scaled orthographic cameras as concluded in other calibration studies. Thus, it appears that their consideration as parameters creates unnecessary degrees of freedom which are vulnerable to noise. Due to the lack of robustness, these methods yield an impractical performance. Nevertheless, it has to be stated that for some special sets of feature points, good results are possible. Because this study is limited to the scenario of short image sequences that allow fast and accurate measurements, the behaviour of these cost functions for larger amounts of SEM images is beyond the scope. For the orthographic and scaled orthographic factorization method, it is expected that three image sequences yield equivalent results to the evaluated four image case. The interested reader is referred to Reference [45] where especially the magnitude of the tilt angles was identified to have an impact on the results provided by the orthographic factorization method.

To improve the preconditions for the dense matching algorithms and to obtain better rectification results, it was demonstrated that the consideration of the images’ individual scaling factors has a beneficial impact on the rectification result. The outlined computation of a similarity transform outperformed the approach linked to the purely orthographic model allowing us to reduce the deviations to the perfectly rectified state. Based on the rectified images, meaningful representations of the surface structure can be reconstructed from just two images. Nevertheless, the general limitation of photogrammetric approaches, solving the correspondence problem in smooth and featureless surface parts, exists as well for SEM images. Even though the applied SGBM generated robust disparity maps for the considered stereo-pairs, the application of extended matching algorithms could help to enhance the accuracy of the provided disparity maps.

The final registration step allows to combine short image sequences separated by large view alterations. This allows to reduce the overall necessary amount of images to reconstruct large parts of the investigated sample. Well distributed correspondences between these images are not necessary because they are only used to determine an initial rough alignment. In addition, affine invariant feature algorithms like ASIFT allow to match features even if the view alteration is very high. The good alignment of the clouds reconstructed from different image sequences after applying the Trimmed ICP algorithm demonstrates that the overlapping surface parts majorly coincide.

Although the direct results of the factorization methods can yield accurate results close to the CLSM data in most scenarios, adding subsequent refinement steps to the reconstruction routine should be considered. It is feasible to apply bundle adjustment algorithms that can be adjusted to the properties of the processed image sequence. For example, in Reference [46], a bundle adjustment with inner constraints for the scaled orthographic camera is presented which could improve the obtained results even further.

## 5. Conclusions

This study demonstrates which accuracies can be expected for reconstructions from SEM images based on factorization approaches to recover the parameters of the affine camera model. By outlining a reconstruction routine, a detailed overview of modelling the SEM’s image properties under affine projection is given. The consideration of individual scaling factors for every image that can be affected by the specimens motion during the tilting are beneficial in the lower magnification range. Otherwise, the purely orthographic camera model seems to be less susceptible to noise. The computation of a rectifying similarity transform based on affine epipolar geometry allowed to enhance the results compared to the approach based on the purely orthographic camera model. In combination with dense matching algorithms and the presented registration scheme, large parts of the specimen can be reconstructed with an accuracy close to CLSM measurements. Although some limitations regarding the affine camera model and the dense matching algorithms were identified, the promising results form the basis for more studies dealing with surface structure analyses applying the SEM and the presented reconstruction routine.

## Figures and Tables

**Table 1:** RMSE given as the symmetric epipolar distance in px${}^{2}$ for the fundamental matrix $F_{R}$ of a rectified stereo-pair. The angle $\Delta \varphi_{S,Y}$ denotes the intended stage tilting difference for each stereo-pair with a manually adjusted scan rotation. $\Delta \varphi_{S,R}$ stands for an additional rotation of the stage in the tilted position. (Spheres, DEP I: 2000 × 2000 px; Pebble Stone, DEP II: 1000 × 1000 px).

Method	Sphere 1 mm	Sphere 300 µm	Sphere 300 µm	Pebble Stone	DEP I	DEP II
*m*	76	544	296	630	963	463
mag	60	200	200	150	400	1000
$\Delta \varphi_{S,Y};\varphi_{S,R}$	10${}^{\circ };0^{\circ }$	$5^{\circ };0^{\circ }$	$15^{\circ };0^{\circ }$	$10^{\circ };0^{\circ }$	$10^{\circ };0^{\circ }$	$10^{\circ };15^{\circ }$
None	2.1220	973.54	907.54	1.7494	7.9539	46303
Rigid	0.9592	0.7864	1.1596	0.4086	0.3235	2.2329
Similarity	0.5992	0.6260	0.6179	0.3639	0.3167	0.7635

**Table 2:** Recovered parameters for a four image sequence of the 300 µm sphere. $r_{\mathrm{LSQ}}$ denotes the radius of the spherical LSQ fit to the measurement result of the applied method based on 72 feature matches. ($r_{\mathrm{sphere}}=150\pm 2.5$ µm, sphericity = 0.625 µm).

Method	$r_{\mathrm{LSQ}}$ in µm	RMSE in µm	$k_{1}$	$k_{2}$	$k_{3}$	$k_{4}$	*c*	*s*
Orthographic (OR)	146.70	0.8748	1	1	1	1	1	0
Scaled Orthographic (SC)	151.93	0.8247	1	0.9992	0.9984	0.9999	1	0
Weak Perspective (WP)	149.49	3.6932	1	0.9993	0.9984	0.9984	1.1632	0
Affine (A)	203.16	20.1177	1	0.9994	0.9984	0.9999	0.5879	−0.1032
Weak Perspective (c=1)	155.54	0.8531	1	0.9993	0.9984	0.9984	1	0
Affine (c=1,s=0)	151.97	2.8265	1	0.9994	0.9984	0.9999	1	0
CLSM	150.86	0.5251	-	-	-	-	-	-

**Table 3:** Rotation angles for the different factorization methods and the four image sequence of the sphere with a radius of 150 ± 2.5 µm. The results are given selecting the first view as a reference view with $\left(\varphi_{X,1},\varphi_{Y,1},\varphi_{Z,1}\right)=\left(0^{\circ },0^{\circ },0^{\circ }\right)$. The tilt motion was performed with $\Delta \varphi_{Y}\approx 5^{\circ }$.

Method		$\varphi_{X,2}$	$\varphi_{Y,2}$	$\varphi_{Z,2}$		$\varphi_{X,3}$	$\varphi_{Y,3}$	$\varphi_{Z,3}$		$\varphi_{X,4}$	$\varphi_{Y,4}$	$\varphi_{Z,4}$
Orthographic		0.12${}^{\circ }$	4.67${}^{\circ }$	0.03${}^{\circ }$		−0.50${}^{\circ }$	9.36${}^{\circ }$	−0.03${}^{\circ }$		−0.51${}^{\circ }$	14.03${}^{\circ }$	−0.02${}^{\circ }$
Scaled Orthographic		0.13${}^{\circ }$	5.03${}^{\circ }$	0.03${}^{\circ }$		−0.54${}^{\circ }$	10.08${}^{\circ }$	−0.04${}^{\circ }$		−0.55${}^{\circ }$	15.11${}^{\circ }$	−0.03${}^{\circ }$
Weak Perspective		0.16${}^{\circ }$	5.26${}^{\circ }$	0.03${}^{\circ }$		−0.65${}^{\circ }$	10.51${}^{\circ }$	0.02${}^{\circ }$		−0.66${}^{\circ }$	15.68${}^{\circ }$	0.01${}^{\circ }$
Affine		−0.44${}^{\circ }$	4.99${}^{\circ }$	0.01${}^{\circ }$		−1.36${}^{\circ }$	9.99${}^{\circ }$	0.11${}^{\circ }$		−1.91${}^{\circ }$	14.99${}^{\circ }$	0.22${}^{\circ }$

**Table 4:** Scaling factors for the images of the considered sequences given by the scaled orthographic (SC) factorization method and the deviations to the orthogonal projection scenario. Intrinsic parameters *c* and *s* provided by the cost functions for the weak perspective (WP) and general affine (A) camera.

Image Sequence	Mag.	m	$k_{2,\mathrm{SC}}$	$k_{3,\mathrm{SC}}$	$k_{4,\mathrm{SC}}$	$\sum_{f=1}^{n=4}\left|k_{f,\mathrm{SC}}-1\right|$	$c_{\mathrm{WP}}$	$c_{A}$	$s_{A}$
Chalked Sphere I	60	207	1.0025	1.0024	1.0012	0.0064	1.1498	0.6543	0.0193
Chalked Sphere II	60	50	1.0024	1.0023	1.0097	0.0157	0.7606	0.3027	0.0464
Gravel Particle	150	395	0.9996	0.9987	0.9977	0.0042	0.7067	0.0049	−0.0225
Sphere 300 µm	200	72	0.9992	0.9984	0.9999	0.0025	1.1632	0.5879	−0.1032
DEP Agglomerate	400	400	0.9989	1.0004	0.9987	0.0027	0.9975	0.1658	−0.0119

**Table 5:** Deviations of the estimated tilt angles to the expected manually set stage angles for the image sequences in that the results of the orthographic and scaled orthographic factorization methods differed significantly. $e_{\Delta \varphi_{Y,f}}$ is defined as $|\Delta \varphi_{Y,f}-5^{\circ }|$.

Image Sequence	Method		$e_{\Delta \varphi_{Y,2}}$		$e_{\Delta \varphi_{Y,3}}$		$e_{\Delta \varphi_{Y,4}}$		$\sum_{f=1}^{n=4}e_{\Delta \varphi_{Y,f}}$
Sphere 300 µm	OR		0.33${}^{\circ }$		0.64${}^{\circ }$		0.97${}^{\circ }$		1.97${}^{\circ }$
	SC		0.03${}^{\circ }$		0.08${}^{\circ }$		0.11${}^{\circ }$		0.22${}^{\circ }$
Chalked Sphere I	OR		0.42${}^{\circ }$		0.93${}^{\circ }$		1.71${}^{\circ }$		3.06${}^{\circ }$
	SC		0.12${}^{\circ }$		0.17${}^{\circ }$		0.01${}^{\circ }$		0.30${}^{\circ }$
Chalked Sphere II	OR		0.81${}^{\circ }$		1.55${}^{\circ }$		1.94${}^{\circ }$		4.03${}^{\circ }$
	SC		0.02${}^{\circ }$		0.11${}^{\circ }$		0.56${}^{\circ }$		0.69${}^{\circ }$

## Abbreviations

The following abbreviations are used in this manuscript:

2D	two-dimensional
3D	three-dimensional
A	affine
C2C	cloud-to-cloud
CLSM	confocal laser scanning microscope
DEP	diesel exhaust particulate
DOF	depth of field
EVT	Everhart Thornley
ICP	iterative closest point
LSQ	least squares
MLESAC	maximum likelihood estimation sample consensus
OA	optical axis
OR	orthographic
GM	guided matching
RANSAC	random sample consensus
RMSE	root-mean-square error
SC	scaled orthographic
SEM	scanning electron microscope
SGBM	semi global block matching
SIFT	scale invariant feature transform
WD	working distance
WP	weak perspective
